# Density-dependence and within-host competition in a semelparous parasite of leaf-cutting ants

**DOI:** 10.1186/1471-2148-4-45

**Published:** 2004-11-14

**Authors:** William OH Hughes, Klaus S Petersen, Line V Ugelvig, Dorthe Pedersen, Lene Thomsen, Michael Poulsen, Jacobus J Boomsma

**Affiliations:** 1 Department of Population Biology, Institute of Biology, University of Copenhagen, Universitetsparken 15, 2100 Copenhagen, Denmark; 2 Department of Ecology, The Royal Veterinary and Agricultural University, Thorvaldsensvej 40, 1871 Frederiksberg C, Denmark; 3 School of Biological Sciences, A12, University of Sydney, Sydney, N.S.W. 2006, Australia

## Abstract

**Background:**

Parasite heterogeneity and within-host competition are thought to be important factors influencing the dynamics of host-parasite relationships. Yet, while there have been many theoretical investigations of how these factors may act, empirical data is more limited. We investigated the effects of parasite density and heterogeneity on parasite virulence and fitness using four strains of the entomopathogenic fungus, *Metarhizium anisopliae *var. *anisopliae*, and its leaf-cutting ant host *Acromyrmex echinatior *as the model system.

**Results:**

The relationship between parasite density and infection was sigmoidal, with there being an invasion threshold for an infection to occur (an Allee effect). Although spore production was positively density-dependent, parasite fitness decreased with increasing parasite density, indicating within-host scramble competition. The dynamics differed little between the four strains tested. In mixed infections of three strains the infection-growth dynamics were unaffected by parasite heterogeneity.

**Conclusions:**

The strength of within-host competition makes dispersal the best strategy for the parasite. Parasite heterogeneity may not have effected virulence or the infection dynamics either because the most virulent strain outcompeted the others, or because the interaction involved scramble competition that was impervious to parasite heterogeneity. The dynamics observed may be common for virulent parasites, such as *Metarhizium*, that produce aggregated transmission stages. Such parasites make useful models for investigating infection dynamics and the impact of parasite competition.

## Background

In most models of host-parasite dynamics, the parasites are considered as part of discrete infections, involving only a single parasite. However, as the transmission stages of parasites tend to be clustered, the majority of host-parasite interactions will rather involve multiple parasite individuals. This is particularly the case for those parasites that exhibit a semelparous life-history, releasing all their transmission propagules in a single event that normally coincides with host death [1,2]. In the same models, transmission is assumed to follow the mass action principle, *βSI*, where *S *and *I *are the densities of susceptible and infected individuals respectively and *β *is a constant describing the probability of infection [3]. Thus the probability of a host becoming infected per unit time will be directly proportional to the number of parasites that it encounters. Host-parasite interactions will also often involve multiple genotypes of parasites, further complicating their dynamics [4,5].

The co-occurrence of multiple parasites within a single host makes within-host competition between the parasites inevitable. Hosts represent limited resources and there is a carrying capacity for the total biomass of parasites that the host resources can support. The within-host growth of the parasites will thus normally be density-dependent, decreasing as the carrying capacity is approached [6,7]. Interactions between different parasite genotypes are often considered to result in increased virulence, but have also been predicted to lead to decreased virulence depending on the dynamics [4,8-11]. The outcome of interactions will depend critically on both the scale of competition and the relatedness of the parasites involved, with higher relatedness selecting for reduced competition, or even cooperation, and more local competition offsetting this [12-14]. The nature of the within-host competition has further been characterized as a continuum between two extremes: superinfections, which exhibit contest competition with the most virulent parasites eliminating those less virulent, and coinfections, where the parasites differ little in virulence and resources end up being shared amongst individuals via scramble competition (or in its purest form by parasites exploiting different within-host niches and not competing at all) [8,15,16]. The mechanism of competition can be: (1) exploitation, with parasites competing for resources, (2) interference, with parasites, for example, producing antagonistic compounds, or (3) apparent, being mediated by the host immune system [5]. Exploitation competition is inevitable whenever parasites do not have completely separate niches within the host, while interference competition via antagonistic compounds drives, for example, the interaction between strains of entomopathogenic bacteria that produce bacteriocins [17]. Apparent competition has been argued to be the most important type [4], and can occur even in invertebrates with their less complex immune systems. For example, malaria parasites suppress the immune response of their mosquito vector [18], and various entomopathogenic fungi have been shown to produce immunodepressant compounds [19-23].

In spite of the fundamental importance of interactions between different strains of parasites, empirical studies of mixed infections are relatively rare and a number of them have produced results that conflict with the theoretical predictions [5,24]. Most studies have found either that mixed infections are more virulent than single infections [25-29], or that virulence equalled that of the most virulent strain [23,25,30-32]. Interactions can be more complex though, and may also depend on environmental conditions, host genotype, or on the parasite genotypes involved [25,32-35]. For example, the virulence of bacteriocin-producing bacteria matches the winning strain only when one strain can kill the other, but is reduced compared to single infections when both strains can kill each other [17]. Furthermore, consideration of virulence only does not provide a full picture of the complexity of the interaction. The production of transmission stages may be increased [26-28,30] or decreased [31] during mixed infections, and may also depend upon the order of infection [32]. Although the finding that virulence matched that of the most virulent strain might suggest that that strain has outcompeted the less virulent strain, in some studies the transmission stages produced were from the both strains [29,31,33], or even entirely from the less virulent strain [23].

Here we examine the infection dynamics of the parasite *M. anisopliae *var. *anisopliae *(Metschnikoff) (Deuteromycotina: Hyphomycetes) in the leaf-cutting ant *Acromyrmex echinatior *Forel (Hymenoptera: Formicidae: Attini). *M. anisopliae *var. *anisopliae *is a generalist entomopathogenic fungus that is known to infect leaf-cutting ants [36-41], as well as many other insects. Infection may be from sporulating cadavers or from spores dispersed in the soil. *Metarhizium *spores have a tendency to remain attached to one another, making even dispersed spores likely to be clustered, and soil has been estimated to contain as many as 1,000 to 50,000 spores g^-1 ^[41]. Spores germinate and penetrate directly through the host cuticle without first growing over the surface of it as some other fungi do. Host individuals will thus often be infected by multiple parasite propagules. In addition, the group-living life-style of leaf-cutting ants, as with other social insects, makes them especially prone to being exposed to multiple infections under natural conditions [42]. Inside the host, the parasite produces blastospores and then hyphal bodies that release immunodepressant and antibiotic toxins [19,21,22]. After a period of time the host dies by some combination of the depletion of its resources due to the parasite infection, direct invasion of tissues by hyphae or the action of the parasite's toxins [21], and the parasite sporulates shortly after this. *Metarhizium *thus has a semelparous life-history and is an 'obligate killer' [1], producing transmission stages only after host death. Such parasites represent excellent models for studying infection-growth dynamics because the rate of successful infections equates exactly to host mortality, the time of host death will relate to the number of hyphal bodies within the host and thus acts as a gauge of within-host growth, and because the lifetime reproductive output of the parasite is represented entirely by the spores produced upon host death in contrast to other parasites that produce transmission stages continually [1]. Yet while applied studies using *Metarhizium *are common, fundamental studies of its infection dynamics are rare.

There is a diversity of *M. anisopliae *var. *anisopliae *strains near leaf-cutting ant nests in Panama [41], indicating the potential for within-host competition between multiple parasite genotypes. We established and compared the dynamics of within-host competition in this system at two levels. We first investigated competition between parasites of the same genotype by establishing whether the infection rate of *M. anisopliae *var. *anisopliae *adhered to the mass action principle and whether parasite growth and fitness was density-dependent. We then examined whether the infection dynamics were consistent between different strains of parasite, and how the dynamics were affected by intraspecific within-host competition involving multiple parasite genotypes. We used two Panamanian strains that had had the opportunity to coevolve with *A. echinatior *and one exotic strain that had never previously encountered the host. A number of studies suggest that exotic strains are less competitive during within-host interactions than are native strains that have coevolved with the host [43-48]. We define parasite virulence to be parasite-induced host mortality as measured by case mortality and time-of-death. This differs from the instantaneous mortality rate used in many models but has been argued to be a more suitable measure of virulence [49]. Note that because the ant hosts were adult individuals that do not grow any further, the potential advantage to an obligate killer parasite of delaying host death to allow further host growth before semelparous reproduction [1], will not apply in this system.

## Results

### Experiment 1: intra-strain competition

**Figure 1:**
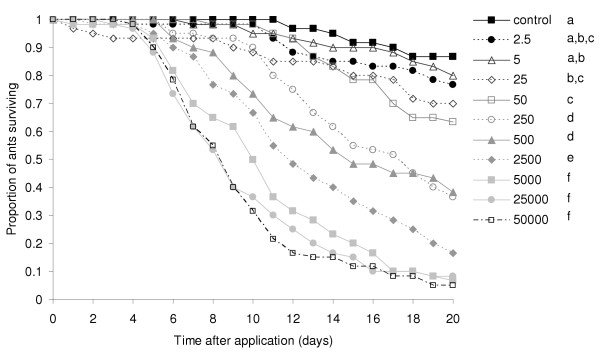
Survival of ants in Experiment 1 treated with serial doses (spores/ant) of *M. anisopliae *var. *anisopliae *isolate KVL 02–56 or a control solution of 0.05% Triton-X (n = 60). Different letters indicate doses whose survival distributions differed significantly.

**Figure 2:**
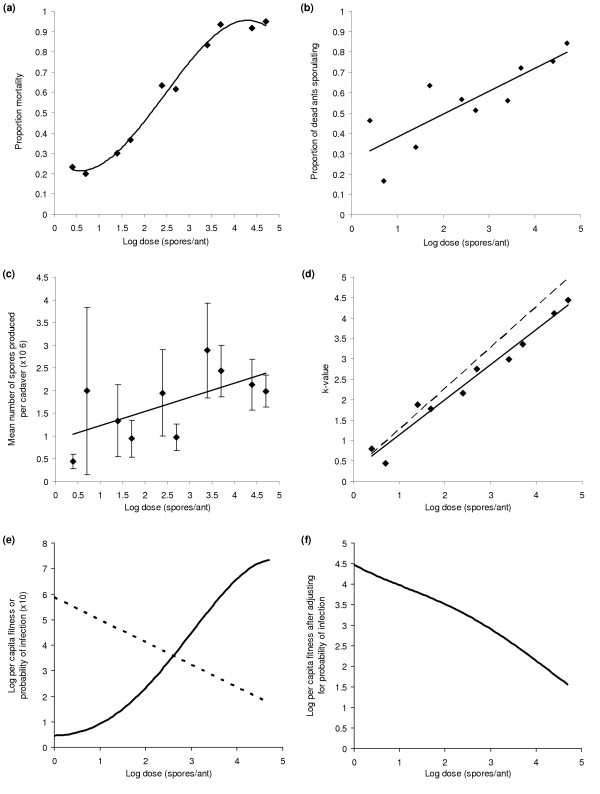
Dose relationships for ants in Experiment 1 treated with serial doses of *M. anisopliae *var. *anisopliae *isolate KVL 02–56 or a control solution of 0.05% Triton-X. (a) Mortality at end of experiment (*y *= -0.021*x*^3 ^+ 0.144*x*^2 ^- 0.054*x *+ 0.171, *r*^2 ^= 0.986). (b) Proportion of dead ants sporulating (*y *= 0.113*x *+ 0.269, r^2 ^= 0.700). (c) Mean number of spores (± SE) produced per sporulating ant (*y *= 0.3692*x *+ 0.6692, *r*^2 ^= 0.4081). (d) *k*-values for ants in Experiment 1 treated with serial doses of *M. anisopliae *var. *anisopliae *isolate KVL 02–56 (*y *= 0.809*x *+ 0.468, *r*^2 ^= 0.976). The dashed line has a slope of 1 and is included for comparison. (e) Per capita fitness (dashed line) and probability of infection (solid line; calculated by multiplying the probability of death and of sporulation if death occurs). (f) Per capita fitness after adjusting for the probability of infection.

The dose of *Metarhizium anisopliae *var. *anisopliae *spores applied had a significant effect on ant mortality (Wald = 131.1, d.f. = 10, P < 0.0001) (Figure 1). The colony of origin did not affect either the dose-response relationship (Wald = 13.2, d.f. = 10, P = 0.213) or mortality overall (Wald = 1.56, d.f. = 1, P = 0.212). The mortality caused by the lowest two doses did not differ significantly from that in the controls (Figure 1). There was also no significant difference in mortality between the highest three doses because mortality was close to 100% at all these doses. The dose-mortality relationship consequently followed a sigmoidal pattern (F_3,7 _= 162.5, P < 0.0001; Figure 2a).

There was no effect of the time between death and the assessment of spore production upon either the proportion of cadavers sporulating (Wald = 1.97, d.f. = 1, P = 0.16) nor the number of spores produced (F_1,127 _= 1.13, P = 0.289). This supports the assumption that sporulation was complete at the time of assessment and also indicates that parasites that took longer to kill their host did not produce more spores. The proportions of cadavers sporulating were significantly less than expected based upon the number of ants estimated (from the control data) to have died from other causes (F_1,17 _= 8.55, P = 0.0095). This indicates that certain ants killed by *Metarhizium *failed to sporulate. Both the proportion of ant cadavers sporulating (Wald = 19.4, d.f. = 9, P = 0.022) and the mean number of spores produced per sporulating cadaver (F_9,127 _= 1.97, P = 0.048) increased significantly with dose (Figures 2b and 2c). However the increase in spore production was relatively small, only doubling over the full range of doses examined. When the spore numbers produced were used to calculate *k*-values (which assess the density-dependence of growth, with a zero value indicating that spore production increases proportionally to dose and positive values indicating spore production is less than proportional to dose), it was found that *k *increased significantly with dose (F_1,9 _= 231.1, P < 0.0001; Figure 2d). The slope of the relationship (0.8) was significantly less than 1 (t = 2.42, d.f. = 8, P = 0.042). Correspondingly, the per capita fitness of the parasite decreased linearly with dose, while, in contrast, the probability of infection (calculated by multiplying the probability of death by the probability of a cadaver sporulating) increased sigmoidally (Figure 2e). By multiplying these two variables, the overall fitness of the parasite can be calculated, and can be seen to decrease more or less linearly with dose (Figure 2f).

### Experiment 2: inter-strain competition

**Figure 3:**
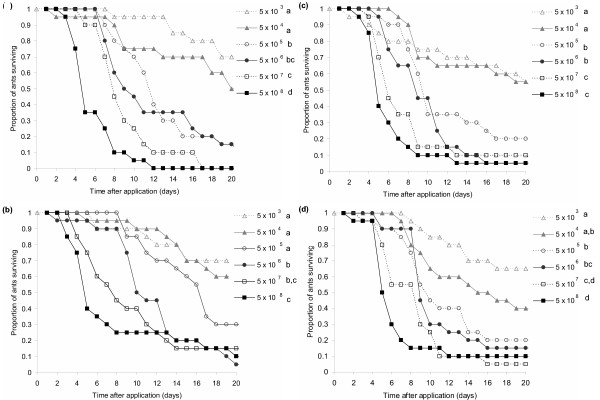
Survival of ants in Experiment 2 treated with either *Metarhizium anisopliae *var. *anisopliae *isolate (a) KVL 02–73, (b) KVL 02–72, (c) Ma275 or (d) a mixture of all three. Different letters indicate doses whose survival distributions differed significantly.

Ant survival was affected significantly by the concentration of *M. anisopliae *var. *anisopliae *spores (Wald = 186.0, d.f. = 5, P < 0.0001), the strain (Wald = 9.42, d.f. = 3, P = 0.024), and also the colony of origin (Wald = 13.5, d.f. = 4, P = 0.009). There were no significant interactions between strain and concentration (Wald = 11.8, d.f. = 15, P = 0.681). Mortality was generally positively correlated with dose, although the significance of pairwise differences between doses did vary somewhat between strains (Figure 3). In none of the strains was there any difference in survival between the lowest two doses, and, with the exception of strain 02–73, there was also no difference in survival between the highest two doses. Overall, ant survival was greatest when treated with the allopatric Ma275 strain, followed by the 02–73 strain (obtained from a soil sample at the collection site), from which it did not differ significantly (Breslow statistic = 2.45, d.f. = 1, P = 0.118). Survival of ants treated with Ma275 was significantly greater than of those treated with either the 02–72 strain (obtained from an *Atta *worker) (Breslow statistic = 8.36, d.f. = 1, P = 0.004) or the mixture of all three isolates (Breslow statistic = 5.00, d.f. = 1, P = 0.025) (Figure 3). The survival distributions of ants treated with the different strains did not otherwise differ significantly (02–72 vs. mixture: Breslow statistic = 0.84, d.f. = 1, P = 0.360; 02–73 vs. mixture: Breslow statistic = 0.26, d.f. = 1, P = 0.613; 02–72 vs. 02–73: Breslow statistic = 2.04, d.f. = 1, P = 0.153).

**Figure 4:**
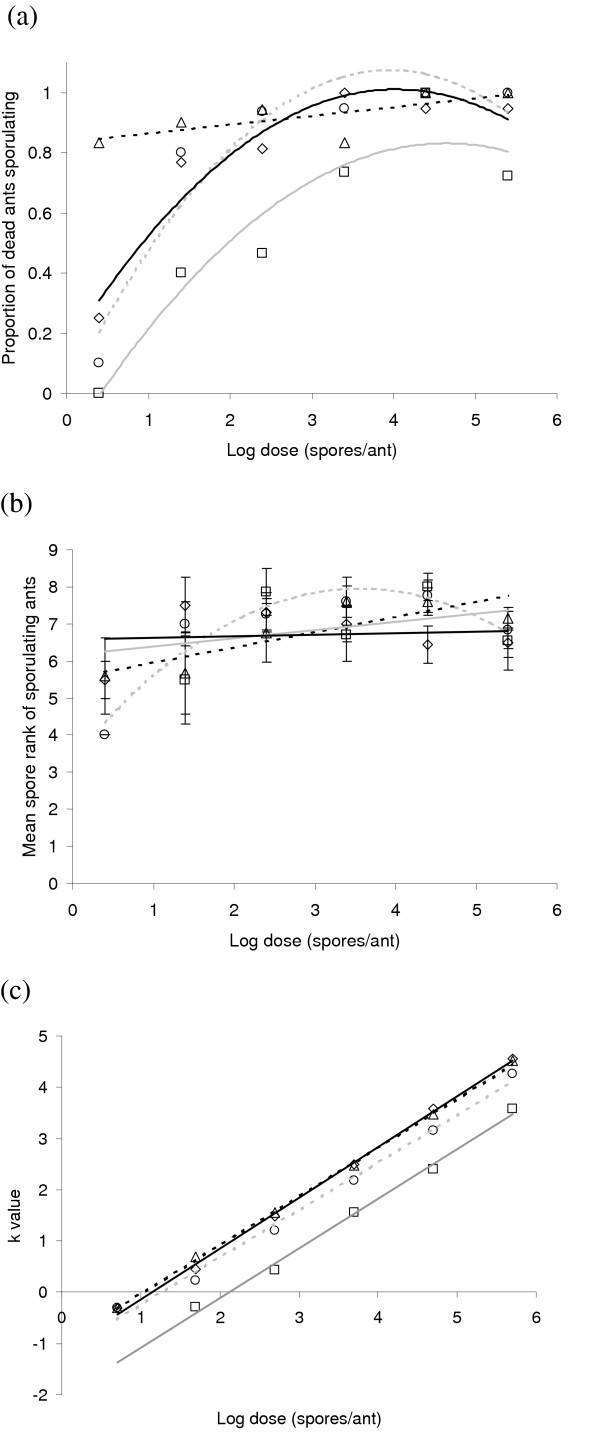
Dose relationships and lines of best fit for ants in Experiment 2 treated with either *Metarhizium anisopliae *var. *anisopliae *isolate KVL 02–72 (circles; grey, dashed line), KVL 02–73 (triangles; black, dashed line), Ma275 (squares; grey, solid line) or a mixture of all three (diamonds; black, solid line). (a) Proportion of dead ants sporulating. Lines of best-fit are: KVL02–72: *y *= -0.0686*x*^2 ^+ 0.5434*x *- 0.0014, *r*^2 ^= 0.8983, P = 0.032; KVL 02–73: *y *= 0.0292*x *+ 0.8339, *r*^2 ^= 0.5182, P = 0.107; Ma275: *y *= 0.1623*x *+ 0.0839, *r*^2 ^= 0.7699, P = 0.030; mixture: *y *= -0.0532*x*^2 ^+ 0.4286*x *+ 0.1476, *r*^2 ^= 0.9291, P = 0.019. (b) Spore ranks of sporulating ants. Lines of best-fit are: KVL02–72: *y *= -0.358*x*^2 ^+ 2.5553*x *+ 3.391, *r*^2 ^= 0.9148, P = 0.025; KVL 02–73: *y *= 0.411*x *+ 5.5392, *r*^2 ^= 0.7212, P = 0.032; Ma275: *y *= 0.222*x *+ 6.1677, *r*^2 ^= 0.116, P = 0.575; mixture: *y *= 0.1261*x*^3 ^- 1.2959*x*^2 ^+ 3.7405*x *+ 4.265, *r*^2 ^= 0.9646, P = 0.833. (c) *k*-values. Lines of best-fit are: KVL02–72: *y *= 0.93*x *- 1.18, *r*^2 ^= 0.992, P < 0.0001; KVL 02–73: *y *= 0.953*x *- 0.982, *r*^2 ^= 0.999, P < 0.0001; Ma275: *y *= 0.972*x *- 2.06, *r*^2 ^= 0.995, P = 0.0002; mixture: *y *= 0.991*x *- 1.127, *r*^2 ^= 0.998, P < 0.0001.

Although the proportion of ant cadavers sporulating was consistently high (>80%) in strain 02–73 and was positively correlated with dose in the other strains (Figure 4a), the interaction between strain and dose was nonsignificant (Wald = 8.92, d.f. = 15, P = 0.882), as were both the main effects (strain: Wald = 2.81, d.f. = 3, P = 0.422; dose: Wald = 5.49, d.f. = 5, P = 0.359). Spore production from the sporulating cadavers was estimated by the ranking method described. It was found to be unrelated to the lag-time between death and ranking (F_1,270 _= 0.489, P = 0.485) and to increase slightly with dose in all treatments (F_5,270 _= 2.42, P = 0.036; Figure 4b). The relationship between spore production and dose did not differ between strains (F_14,270 _= 1.12, P = 0.342) and the strains also did not differ overall (F_3,270 _= 0.19, P = 0.903). The *k*-values for all three strains were positively correlated with the number of spores applied (F_1,15 _= 3612.9, P < 0.0001) and had similar slopes (F_3,15 _= 0.78, P = 0.523; Figure 4c). The values for Ma275, though, were significantly lower than for the other strains (F_3,15 _= 13.71, P = 0.0001).

## Discussion

Parasite virulence, as measured by host mortality, was found to show a clear density-dependent pattern. This has also been recorded previously in *Metarhizium *[e.g. [50-53]] and other parasites [6,7,54]. Rather than being a linear relationship though, the pattern was sigmoidal. Mortality was not increased significantly by further increases in dose beyond 1 × 10^7 ^spores per ml. In addition, the mortality caused by doses of 1 × 10^4 ^spores per ml or less did not differ from that of ants treated with the control solution (Figure 2a). All parasite-induced mortality was expected to have occurred by twenty days after treatment, even at the lower doses, and this assumption was supported by the levelling out of mortality seen in both experiments. It follows from this that the infection rate of *Metarhizium *is directly represented by the host mortality rate after twenty days. The lack of a difference in mortality between ants treated with the two lowest doses and the control ants suggests the occurrence of an Allee effect, with an invasion threshold for infection to be successful [54]. Such an effect has also been evidenced in some other studies of *Metarhizium *[e.g. [50,53]], and is a subtle effect that will only be detected when a sufficient range of doses is tested. Many models of host-parasite dynamics are based upon the mass-action principle, under which the infection rate is a linear function of the density of parasites a host individual encounters [3]. The sigmoidal pattern observed in the *Metarhizium*-*Acromyrmex *system indicates that, here at least, this principle applies only at intermediate doses.

The Allee effect most probably relates to the effectiveness of the host defences against parasites. Leaf-cutting ant defences, as with most insects, consist of 'first-line' defences involving grooming and the secretion of antibiotic compounds on to the cuticle, and 'second line' defences based upon the cellular and humoral immune responses [37,40,55-58]. Spores will interact independently with the first line defences, the immune response may be saturated if it has to defend against very high numbers of parasites. This effect will be exacerbated by the toxins that the hyphae of many *Metarhizium *strains produce to incapacitate the immune system [19,21,22,59]. Greater doses of infecting spores will more quickly produce a larger pool of mycelium, which will produce greater quantities of these toxins and thus make the immune system decreasingly capable of mounting an effective response.

Neither the probability of a cadaver sporulating nor the number of spores it produced were related to the time of host death, indicating both that parasites did not gain increased spore production by taking longer to kill the host, and that spore production was complete at the time of assessment. Both spore production and the proportion of cadavers sporulating were positively correlated with the within-host density of the parasite. However, the efficiency of the conversion of host biomass into parasite propagules was negatively density-dependent, as demonstrated by the *k*-values. The number of spores produced was less than proportional to increases in the dose of parasites applied, indicating the occurrence of density-dependent parasite growth and within-host competition. Although this competition was between genetically identical parasites in the first experiment, it still resulted in a reduction in parasite fitness because of the less efficient use of host resources. Were spore production to be unaffected by, or even inversely related to dose, as found in a study with *Beauveria *[60], then this negative effect on fitness would be even greater.

It is important to note that this is based only upon the cadavers that sporulated. As both the proportion of cadavers that sporulated and the number of ants that died increased with dose, there is a trade-off for the parasite (Figure 2e). Higher doses will result in more hosts dying and producing parasite spores, but where cadavers do sporulate, the per capita spore production will decrease as dose increases. Interestingly, this trade-off resolves itself such that increases in dose always result in a decrease in fitness (Figure 2f). Although low numbers of parasite spores have only a small chance of successfully infecting a host, the strong effect of within-host competition makes dispersal the best strategy. This is even without taking into account the probability of at least one spore encountering a host, which will also be substantially increased by spores dispersing (and thus having multiple chances to encounter a host per unit time), rather than staying in a single aggregation (and having only a single chance per unit time to encounter a host). In other studies of obligate killer parasites, even stronger effects of within-host competition between propagules of the same parasite clone have been found [7,60]. It therefore seems likely that these dynamics may be broadly similar for most semelparous, obligate killer parasites, and that maximum dispersal is the best strategy for these parasites, as well as any others that exhibit strong within-host competition.

When the different strains of *M. anisopliae *var. *anisopliae *were compared in the second experiment, they were found to differ in their virulence. Interestingly, it was the exotic strain (Ma275), originating from a different geographical location and from a different host order (Lepidoptera) that had the lowest virulence, and the strain isolated from a leaf-cutting ant worker (KVL 02–72) that had the highest. Many studies have found *Metarhizium *strains to vary in virulence [e.g. [53,61]]. Differences in strain virulence may in part be due to the variation in the production of destruxins that occurs between strains, but other virulence factors are undoubtedly also important [59]. As virulence is defined here as parasite-induced host mortality, the differences between strains could also be due to differences in the proportions of spores germinating and penetrating into the host rather than differences in within-host growth [62,63]. Although all strains had similarly high germination rates on artificial media, the interaction with host cuticle is more complex and involves various antibiotic compounds such as those produced by the metapleural gland [56,64,65]. Differences between strains in their susceptibility to such compounds would seem quite likely. Aside from the differences in virulence, however, the strains did not otherwise differ in their infection dynamics. They showed similar spore production and identical density-dependent growth patterns. The dynamics described above therefore appear to be consistent across strains, at least for those tested here.

A fundamental assumption of most models of host-parasite relationships is that within-host competition is more intense, and results in heightened virulence, when it involves more than one parasite genotype [8-10]. However, in experimental studies virulence has often been found to be unaffected by parasite heterogeneity [24]. This was also the case in the current study, in which the virulence of the mixed infection was the same as that of the most virulent strain in the infection. In addition, the parasite *k*-value vs. dose relationship was identical for the mixed and single infections, indicating that the density-dependent growth patterns were unaffected by host heterogeneity.

There are two possible explanations for the results. The most parsimonious is that the most virulent strain in the mixed infections simply outcompeted the other strains and drove them to extinction within the host. Such superinfection dynamics have previously been suggested for *Metarhizium *and other entomopathogenic fungi [32,66,67] and would be in accord with some models [6]. However other studies have found mixed infections to produce transmission stages from more than one strain of parasite in spite of the overall virulence matching that of the most virulent strain [29,31]. It remains possible that the mixed infections in the current study did not involve any of the strains being competitively excluded and that the spores produced came from all the strains. To distinguish between these possibilities it would be necessary to isolate and sequence monospore cultures from the sporulating cadavers, something would be an interesting objective for future work.

Importantly, the infection dynamics did not differ between the single and mixed infections. The occurrence of within-host competition, whether it involves the production of antagonistic compounds or is mediated by the host's immune system, might be expected to force parasites to divert some resources from growth and the production of transmission stages to producing or coping with competitive mechanisms. While there is some evidence for this [31], the production of transmission stages has been increased during mixed infections in many other studies [26-28,30]. Clearly the outcome will depend upon the particular genotypes involved. If different strains produce antagonistic compounds that are effective against one another, as in the study by Massey et al. [17], then reduced transmission stages can be expected. If they do not, and if they exploit different within-host niches, produce cooperative compounds (such as iron-binding agents [13,14]), or act synergistically to depress the host immune system, then increased transmission stages can be expected. The fact that spore production was the same in single and mixed infections in the current study therefore suggests either that the most virulent strain outcompeted the others without suffering any cost from the competitive interaction, or that the *Metarhizium *strains were engaged in scramble competition with dynamics that are impervious to interactions being inter- or intraclone.

## Conclusions

The importance of within-host competition between parasites is well illustrated by the *Metarhizium*-*Acromyrmex *system studied here. Even though the probability of a successful infection was increased substantially by parasites occurring in aggregations, the effect of competition between parasite propagules of the same clone makes dispersal the best strategy. It seems likely that this is generally true for semelparous parasites. Further investigations of the impact of within-host competition on parasite fitness are needed and should endeavour to establish where on the superinfection-coinfection continuum the interaction lies by identifying which parasites produce transmission stages. The fact that the production of transmission stages of semelparous parasites, such as *Metarhizium*, is concentrated into a single bout, and thus that their lifetime fitness can be readily quantified, makes them excellent models for doing this.

## Methods

### General methodology

Colonies of *A. echinatior *were collected from Gamboa, Panama, and maintained in the lab under controlled conditions (ca. 24°C, 70% RH) on a diet of bramble leaves (*Rubus fruticosus*) and rice grains. For the experimental replicates, large workers (head width 2.1 to 2.4 mm) were removed from their colonies and placed individually in plastic pots (diameter: 2.5 cm, height: 4 cm) where they were maintained at 24°C with an *ad libitum *supply of water and sugar water. A number of isolates of *M. anisopliae *var. *anisopliae *were collected from the vicinity of leaf-cutting ant nests at the same location in Gamboa, Panama, and were cultured as monospore isolates on Sabouraud dextrose agar [41]. Spore (conidia) suspensions were made by flooding agar plates with mature spores with a sterile solution of 0.05% Triton-X and scraping off the spores with a glass rod. The spores were centrifuged and washed three times with sterile 0.05% Triton-X solution with intervening centrifugation steps. The concentration of spores was then quantified using a haemocytometer and diluted to the required concentration. The viability of the spore suspensions was checked by spreading 100 μl of them on to Sabouraud dextrose agar plates and counting the proportions of spores that had germinated after 12–16 hours at 24°C. Spore viability was >95% in all cases.

Ants were treated with *M. anisopliae *var. *anisopliae *by applying 0.5 μl of a spore suspension to their thorax using a micropipette. Spore suspensions were vortexed thoroughly immediately prior to application to ensure spores were fully dispersed. Control ants had 0.5 μl of a 0.05% Triton-X solution applied in the same way. Following application, ant mortality was assessed daily for a period of twenty days. Based on previous work [23,40,56], this time period was judged sufficient to ensure that all parasite-induced mortality had occurred by the end of the experiment. Dead ants were surface sterilised [68], and placed in a petri dish lined with damp filter paper. After the completion of the experiments, the cadavers were left for a further ten days in order to allow full sporulation of the parasite. The level of sporulation on the cadavers was then assessed by one of two methods. In Experiment 1, sporulation was quantified by directly counting the number of spores on the cadavers. The cadavers were placed in individual vials with 1 ml of 0.05% Triton-X solution and vortexed for 1 min to remove the spores into suspension. The concentration of spores in the suspension was then quantified with a haemocytometer. In Experiment 2, sporulation was estimated by examining the cadavers under a binocular microscope and giving each a rank of between 0 (no spores visible) and 10 (cadaver almost completely covered by spores) depending upon the level of sporulation. Based on data collected prior to these experiments, spore ranks estimated in this manner correlate well with the actual number of spores on the cadavers (*y *= 0.159*x *- 0.862, *r*^2 ^= 0.746, Spearman's r = 0.820, N = 73, P < 0.01) and thus provide a reliable estimate of spore production.

### Experiment 1: intra-strain competition

The experiment involved *M. anisopliae *var. *anisopliae *isolate KVL 02–56, which had been isolated from the dump pile of an *Atta colombica *nest in Gamboa, Panama [41]. A spore suspension was made up and serially diluted five-fold to give concentrations from 1 × 10^8 ^to 5 × 10^3 ^spores ml^-1 ^(equivalent to an average of 50,000 and 2.5 spores per ant respectively). Given that soil at the site in Panama has been estimated to contain as many as 1,000 to 50,000 spores g^-1 ^[41], it seems likely that this range encompasses the natural doses that the ants are exposed to. Thirty ants from each of two colonies of *A. echinatior *(Ae47 and Ae109) were treated with each of these doses, or with the control solution, and their survival monitored for twenty days after application. The effect of dose and colony of origin on ant mortality was analysed with a Cox proportional hazard regression model to examine the effect of parasite density (dose) on virulence and infection rate. This incorporates both case mortality and the time of death. Pairwise comparisons of the doses were done using Kaplan-Meir survival analyses and the Breslow statistic. The ant cadavers were left for ten days after the end of the experimental period, in order to allow ample time for all cadavers to sporulate fully. The numbers of cadavers sporulating and the numbers of spores produced by these cadavers were assessed with binary logistic and general linear models respectively. These data were used to calculate *k*-values to assess whether parasite growth was density dependent, as done previously by Ebert et al. [7]:

*k*_i _= log_10 _(M_0 _D_i _/ M_i _D_0_)

where *k*_i _is the *k*-value at dose *i*, *M *is the number of spores produced, *D *is the number of spores applied, and *M*_*0 *_and *D*_*0 *_represent the number of spores produced and applied respectively at the lowest dose tested. A zero value for *k *indicates that the number of spores produced at dose *i *are exactly proportional to the increased dose (so, for example, a doubling of dose results in a doubling of the number of spores produced). The equation gives a positive value for *k *when the number of spores produced at dose *i *are less than proportional to the increase in dose. Zero values for *k *thus indicate that spore production is independent of parasite density, while positive values of *k *indicate that spore production is negatively density dependent.

### Experiment 2: inter-strain competition

To establish whether the dynamics recorded in the first experiment were consistent for different strains of the parasite, three isolates of *M. anisopliae *var. *anisopliae *were compared. These were KVL 02–73 (isolated from soil at the field site from which the ant colonies were collected), KVL 02–72 (isolated from an *Atta colombica *leaf-cutting ant worker), and the strain Ma275 (isolated from *Cydia pomonella *(Lepidoptera: Tortricidae) in Germany). The strains therefore represented a range in terms of their potential coevolution with *A. echinatior*, with the former two being likely to have had some interaction while Ma275 would not previously have encountered *A. echinatior*. Each strain was diluted tenfold and tested at six doses from 5 × 10^8 ^to 5 × 10^3 ^spores ml^-1^. In addition, a mixed spore suspension was made up to examine inter-strain competition. The suspension contained equal numbers of spores of each of the three strains. This was serially diluted and applied at the same doses as the individual isolates. Four ants from each of five colonies of *A. echinatior *(Ae48, 109, 143, 153 and 154) were treated with each dose of each isolate or with the control solution and their survival monitored for twenty days. The survival of the ants was analysed as in the preceding experiment to examine if strains differed in virulence and if the growth of each strain was density-dependent. Ten days after the end of the experimental period, spore production was assessed by ranking each cadaver for the amount of sporulation as described earlier. These ranks were used to estimate the actual number of spores produced and these values were then used to calculate *k*-values. A regression analysis was carried out on these data in order to assess if the growth of each strain was density-dependent and whether the density-growth dynamics differed between strains.

## Authors' contributions

WOHH conceived the study, assisted with the experiments, analysed the results, and drafted and revised the manuscript. KSP, LVU and DP carried out Experiment 1 and KSP also participated in Experiment 2. MP assisted with both experiments. LT assisted with mycological aspects and JJB provided support throughout. All authors contributed to the writing of the manuscript.
